# Intersectional Immunity? Examining How Race/Ethnicity and Sexual Orientation Combine to Shape Influenza Vaccination Among US Adults

**DOI:** 10.1007/s11113-022-09739-x

**Published:** 2022-09-19

**Authors:** Kiana Wilkins

**Affiliations:** Department of Sociology, Rice University, 6100 Main Street, Houston, TX MS-2877005 USA

**Keywords:** Andersen’s healthcare utilization model, Intersectionality, Race/ethnicity, Sexual orientation, Influenza vaccination

## Abstract

Influenza vaccination is a critical preventive healthcare behavior designed to prevent spread of seasonal flu. This paper contributes to existing scholarship by applying an intersectional perspective to examine how influenza vaccination differs across specific intersections of racial/ethnic and sexual identity. Drawing on aggregated state-level data from Behavioral Risk Factor Surveillance System (BRFSS) from 2011 to 2020, I examine how flu vaccination differs across 18 racial/ethnic-by-sexual orientation groups (*N* = 1,986,432). Findings from descriptive analyses and logistic regression modeling demonstrate three key findings. First, it corroborates previous studies of vaccination, finding lower rates of flu vaccination among black adults relative to whites; gays/lesbians vaccinate at higher rates than heterosexuals and bisexuals, with bisexuals reporting lower vaccination relative to both heterosexuals and gays/lesbians. Second, it demonstrates how sexual orientation complicates established patterns between race/ethnicity and vaccination (e.g., influenza vaccination is more racially stratified among heterosexuals, with patterns more variable among gays/lesbians) and how race/ethnicity complicates previous patterns of vaccination by sexual orientation (e.g., Asian bisexuals vaccinate more than both heterosexuals). Third, findings pinpoint identities (e.g., black heterosexuals relative to their white peers and white bisexuals relative to their gay/lesbian peers) most in need of influenza vaccination outreach efforts. Implications for findings suggest that heterosexuals, especially black, may be less likely to vaccinate against influenza thus may need more encouragement from clinicians to vaccinate. Additionally, influenza vaccination should be free for all persons to lessen the barrier of access for this preventative healthcare.

## Introduction

During the 2017–2018 flu season, an estimated 41 million persons in the United States contracted influenza, resulting in 21 million influenza-related medical care visits and approximately 710,000 hospitalizations (CDC, 2022). Influenza is consistently a significant source of US mortality, ranked as the 9th leading cause of death in 2019 (CDC, 2020). Vaccination is the most effective strategy to reduce influenza-related morbidity and mortality, with vaccination recommended for *everyone* over six months of age (CDC) and necessary for both individual and community health (US Department of Health & Human Services, 2022). Because of the substantial health consequences related to this illness, a yearly influenza vaccination is recommended by the Centers for Disease Control and other medical organizations. Influenza vaccination also offers a useful model of understanding disparities in healthcare, as vaccines are administered frequently, no referral is needed, and this intervention requires no follow up care (Fiscella, 2005).

While each flu season generally brings concerns for population health, it is especially worrisome for groups more vulnerable to physical and socioeconomic strains associated with influenza—including selected racial/ethnic and sexual minorities. Mortality rates for various medical conditions are elevated among marginalized groups (Cogburn, 2019; Williams & Sternthal, 2010; Williams et al., 2019a, 2019b), as they are, on average, less able to afford both the economic and bodily costs (Hutchins et al., 2009). Studies have repeatedly documented diminished health and healthcare access among selected racial/ethnic groups, with black, American Indian/Alaska Native (hereafter referred to as AI/AN), and some Hispanics experiencing some of the poorest outcomes (Boen & Hummer, 2019; Cogburn, 2019; Warne & Frizzell, 2014; Williams et al., 2019a, 2019b). Simultaneously, a growing body of scholarship demonstrates disparities in health and healthcare based on sexual orientation (Hafeez et al., 2017; IOM, 2011), with bisexuals experiencing poorer outcomes relative to heterosexual and gays/lesbians (Gorman et al., 2015; Schick, 2012). Less understood is how racial/ethnic and sexual identity combine to shape protective to health behaviors, including flu vaccination. For example, individuals experiencing multiple marginalization on the basis of race/ethnicity and sexual orientation (e.g., black bisexuals) may find themselves in an especially precarious situation of navigating macro-level and micro-level stressors and barriers to preventive healthcare (Harris et al., 2018; Tuthill, Denney and Gorman, 2020).

I explore these issues by utilizing an intersectional framework, examining how flu vaccination varies across specific intersections of race/ethnicity and sexual orientation among US adults. Specifically, I examine data from a sample of adults from 45 states included in the 2011–2020 interview years from Behavioral Risk Factor Surveillance System (BRFSS). In following analyses, I utilize Andersen’s (1995) behavioral healthcare utilization model to examine flu vaccination patterns among individuals across 18 intersections of race/ethnicity and sexual orientation. My analyses compare those who, on average, are in more socially advantaged positions (i.e., white heterosexuals), respective to those who are in least advantaged social positions (e.g., AI/AN bisexuals), and also evaluating those who concurrently occupy advantaged and disadvantaged positions (e.g., black heterosexuals). I examine logistic regression models among the pooled sample and following run models stratified by race/ethnicity. In each set of models, I situate ‘intersectional immunity’ (i.e., overlapping identity characteristics which shape vaccination status) within population health research by controlling for need-based predisposing, enabling, and need-based characteristics (Andersen, 1995).

## Background

Influenza is a consistent contributor to US mortality, typically listed as a top ten contributor to US mortality (CDC, 2020). As mentioned above, vaccination is the most effective strategy to reduce influenza death and illness, with vaccination being recommended for anyone over 6 months of age to prevent disease among individuals and their communities (CDC, 2021a, 2021b, 2021c; US Department of Health & Human Services, 2022). Studies often focus on vaccination among elderly and/or high-risk individuals, rather than larger population-based samples (e.g., Travers et al., 2018). Scholarship using National Health Interview Survey (NHIS) found in 2015–2016, influenza vaccination had increased among adults aged 18–64 (at 1.5%, on average, each year) compared to 2010–2011 (Tian et al., 2019). Although there is evidence for general increases in vaccination among adults aged 18–64 in flu vaccine uptake, racial/ethnic vaccination disparities still persist (Quinn, et al., 2018,2018,2018) and influenza vaccination annual trends by sexual orientation remain largely unexplored, with some evidence of gay/lesbian advantage and a bisexual disadvantage.

Influenza affects individuals in two prominent ways: financially and physically. A recent analysis estimates influenza costs US patients 10 billion annually (Federici, Cavazza, Costa, & Jommi, 2018), with US hospitalizations costing upwards of $7,000 for patients. Racial/ethnic minorities (specifically black, Hispanic, AI/AN, and some ethnic Asian groups) disproportionally hold low-wage, less-flexible, and benefit deficient jobs, making it difficult or impossible to take time off from work for sickness (Phelan & Link, 2015; Williams & Jackson, 2005). Sexual minorities, especially bisexuals, experience a particular disadvantage (IOM, 2011; Gorman et al., 2015), as they report vulnerable economic profiles, suggesting influenza illness may be especially burdensome among selected sexual identity groups. In terms of bodily cost, during the 2009 H1N1 influenza pandemic, AI/AN adults reported the highest influenza-related hospitalizations, followed by Hispanic and black adults (Dee, et al., 2011; Uscher-Pines, et al., 2011). Importantly, hospitalizations indicate influenza’s strong and negative impact on the body.

### Theoretical Frameworks

An intersectional framework offers a critical tool for understanding disadvantaged (and advantaged) health and healthcare access outcomes based on various combinations of identity characteristics (Bowleg, 2012). Intersectionality highlights the potential for poorer outcomes for persons who are both racial/ethnic and sexual minorities, and the potential for health advantage among persons who hold neither a sexual nor a racial/ethnic minority identity. As a perspective, it underscores how identity categories operate as categorical markers that intersect to operate multiplicatively and reflect systems of interlocking oppression and privilege (Bowleg, 2012; Collins, 2016; McCall, 2005). Persons who simultaneously occupy minority identities based on sexuality and race/ethnicity may face both macro-level mechanisms of discrimination (e.g., lower quality healthcare) in addition to micro-levels of discrimination (e.g., low levels of family support, smaller social networks). In addition, this framework is inherently relational. Intersecting power relations allocate personal and political status among dimensions of overlapping identities that have meaningful impact on individual, and group, lived experiences. Understanding the relational aspect of intersectionality is necessary for public health scholars to engage with and intersectional scholarship must treat these categories as jointly rather than additive to ensure that analyses are not ‘flattened’ (Bowleg, 2021). Applying this framework confronts the notion of a single stratified hierarchy, and instead encourages investigation of complex systems of privilege and oppression, for those within the margins and those who profit from the margins (Collins & Bilge, 2016; Yuval-Davis, 2015).

In this paper, I focus on the intersection of race/ethnicity and sexual orientation. Studies have documented select racial/ethnic minority groups (black, Hispanic, AI/AN, and certain Asian groups) endure more chronic life stressors that substantially strain their well-being and are more likely to face institutional constraints (Williams et al., 2010) and consequentially face more limited social networks (Berkman & Glass, 2000). Sexual minorities also face obstacles to accessing healthcare services, although patterns likely differ across specific identity groups (Meyer, 2001; Institute of Medicine [IOM], 2011), with bisexuals, compared to gays/lesbians and heterosexuals, reporting poorer health outcomes (Gorman et al., 2015; Veenstra, 2013). Racial/ethnic identity and sexual orientation are neither separate or simple demographic characteristics and taken together are one avenue to explore with how power and privilege are differentially and interdependently are related to one another (Poteat, 2021). Like all identity characteristics [such as gender, (dis)ability status, nationality, and class], racial/ethnic status and sexual orientation are relational to one another. For example, a black woman lesbian does not have singular dimensions of a black, woman, and lesbian identity, but rather a *black woman lesbian* identity in which identity characteristics cannot be disentangled as they shape the others (Bowleg, 2008). Continuing with Bowleg’s (2008) example of black lesbian women, this group illustrates how persons can be subjected to mutually reinforcing social hierarchies of race, sex/gender, and sexual orientation. Thus, one who is in the margins may be even further marginalized by other identity characteristics (Harris, 2009).

The complexity of intersectionality (Collins & Bilge, 2016; Misra et al., 2021) offers a unique perspective to study influenza vaccination. As stated in the introduction, influenza vaccination is a relatively easy and accessible preventative healthcare behavior to partake in as it requires no referral, may be administered outside of the doctor’s office in places such as pharmacies and grocery stores, and can be cost-effective or free (Fiscella, 2005). To date, sparse scholarship has yet to examine race/ethnicity and sexual orientation as potential overlapping identity axis of inequality and how these axis shape utilization and access of this preventive healthcare manifests. Given influenza is relatively available for most persons the accessibility of influenza vaccination may highlight agentic options persons can engage with to curb the threat of illness (both financial and physical) and also reveal constrained systems of power that make vaccinating against influenza challenging regardless of accessibility. I sum, I choose to focus on race/ethnicity and sexual orientation as these social categories offer a vehicle to analyze how structures intersect to allocate power and privilege to persons based on their intersectional location of matrixes of racial/ethnic and sexual orientation hierarchies, (2) add quantitative literature on understudied overlapping identities, and to (3) how this shapes preventative healthcare utilization (i.e., influenza vaccination).

Due to the focus on influence vaccination (a preventive healthcare behavior), I also draw on Andersen’s (1995) model of healthcare utilization. This model argues healthcare utilization is influenced by predisposing characteristics (mechanisms marking individuals’ social location), enabling characteristics (those emphasizing ability to access and engage with the healthcare system), and need-based characteristics (factors underlining need for healthcare). These predisposing, enabling, and need-based characteristics may confound relationships between race/ethnicity, sexual orientation, and influenza vaccination, and when evaluating influenza vaccination controlling for these factors is necessary in order to detangle potential mechanisms obstructing or facilitating healthcare utilization across groups.

### Race/Ethnicity, Sexuality, Health, and Vaccination

Research has long established racial/ethnic minorities face major barriers to accessing quality healthcare services. Socioeconomic differences across race/ethnicity contribute to health disparities, with Link and Phelan (1995) arguing socioeconomic status (SES) is a fundamental contributor to health inequalities. Phelan and Link (2015) expand their argument to formulate *racism* as a foundational contributor to health inequalities, as SES inequality is a product of racism. Indeed, many scholars have attributed racism as a fundamental cause of disease by documenting lack of access to care and quality health, as well as mechanisms beyond SES factors (e.g., discrimination in delivery of healthcare services, racial biases in providers of healthcare services) harmfully impact physical and mental well-being among racial/ethnic minority groups (Cogburn, 2019; Williams & Sternthal, 2010; Williams et al., 2019a, 2019b). While Phelan and Link (2015) utilize Feagin’s (2006) concept of systemic racism (primarily rooted in a black/white difference), growing evidence finds AI/ANs and Hispanics (although this is complicated by nativity status) face deleterious health outcomes and lack access to healthcare due to racism (Boen & Hummer, 2019; Warne & Frizzell, 2014).

AI/ANs add concerning caveats when examining health inequalities. They are the only race/ethnicity where the US government has an explicit responsibility for providing healthcare for through Indian Health Care (IHS) services (Warne & Frizzell, 2014)—yet despite this, AI/ANs still have relatively bleak health outcomes, including high rates of obesity and chronic health conditions (Epsey, Cobb, Bartholomew, Becker, Haverkamp, & Plescia, 2014; Warne & Frizzell, 2014). Scholarship has also found Hispanics live longer yet harder lives, as their socioeconomic profiles are lower than whites, but their health risks are similar to black adults (Boen & Hummer, 2019). Fiscella and Sanders (2016) detail how racial/ethnic minorities often face powerful structural barriers to primary care, where preventative healthcare is typically administered. In sum, select racial/ethnic minorities (black, AI/AN, and Hispanic) face many barriers to health equity, such as unequal access to quality health and processes of stress and discrimination causing poor health outcomes (Lee, Ayers, & Kronenfield, 2009).

Consequently, it is not surprising that lower influenza vaccination status is found among black, AI/AN, and Hispanic adults, relative to whites. For example, Lu and colleagues (2014) draw on data from both the NHIS and BRFSS and show, encouragingly, non-Hispanic white, non-Hispanic black, and Hispanic adults aged 18–64 had an increase in influenza vaccination in the 2011–2012 flu season relative to the 2007–2008 flu season. However, their findings also show racial/ethnic disparities persist in influenza vaccination despite vaccination increases, with black and Hispanic adults being less likely to vaccinate against influenza, and some racial/ethnic groups reporting higher vaccination status relative to whites (Asians aged 18–49; AI/ANs aged 18–49). Other works confirm black adults remaining less likely to have their seasonal flu vaccines compared to whites (Quinn, et al., 2017; Quinn, 2018; CDC, 2020), and in 2020, AI/ANs were 20% less likely to be vaccinated against influenza than non-Hispanic whites (U.S Department of Health & Human Services, 2022). Using NHIS interview data, Jang and Kang (2021) found that among foreign born and non-Hispanic black respondents were less likely to be vaccinated against influenza relative to US born and non-Hispanic white respondents.

Turning to sexual orientation, sexual minorities report less access to healthcare, lower quality of healthcare, and face barriers due to stigma in healthcare services relative to heterosexuals (IOM, 2011; Dahlamer, Galinsky, Joestl, & Ward, 2016). Bisexuals in particular tend to be more disadvantage and report poorer health outcomes compared to gays/lesbians. Studies show that, on average, SES is lower among bisexuals (IOM, 2011; Schick, 2012) and bisexuals face external factors such as homophobia and biphobia and subsequently face internalized stigma (Ochs, 1996). Compared to heterosexuals, bisexuals report poorer self-rated health and higher rates of mental illness and substance use (Conron et al., 2010; Veenstra, 2013).

Burgeoning evidence also show complex relationships with health status when racial/ethnic and sexual identity are simultaneously considered. Drawing on data from the NHIS, Hsieh and Ruther (2017) examined differences between whites and non-whites and found that despite health insurance increases, sexual minority non-white- men and women report needing to visit to an emergency room, not having health insurance, and delaying care due to cost at higher odds relative to white straight men. In another paper Hsieh and Ruther (2016) found that relative to straight white men, all racial, gender, and sexual minority adults (except for non-white bisexual men) report poorer health outcomes. Furthermore, Tuthill and colleagues (2020) drew on BRFSS data to examine detailed intersections between race/ethnicity, sexual orientation, and selected health outcomes and found ample evidence sexual orientation and race/ethnicity simultaneously shape health status. For example, self-rated health among bisexuals was poorer than heterosexuals and gays/lesbians for all racial/ethnic groups except black men, but patterns for health behaviors (including obesity and smoking) were more complex. Their findings also demonstrated that while bisexuals were more socioeconomically disadvantaged than gays/lesbians and heterosexuals, sexual orientation operated as a weaker stratifying force of economic status among racial/ethnic groups experiencing higher levels of socioeconomic disadvantage (black, Latino, and AI/AN). Overall, this small but growing literature suggests health outcomes may be particularly poor among adults who are both racial/ethnic and sexual minorities (and especially for bisexuals).

To date, relatively few studies focus on sexual minority vaccination, and those that do typically examine vaccinations against sexually transmitted diseases, such as HPV and Hepatitis A and B. Jones and colleagues (2016) found that among LGBT individuals surveyed in Kentucky, 68% of respondents reported receiving the influenza vaccine, and LGBT respondents overall reported higher percentages of vaccination compared to heterosexuals. Srivastav and colleagues (2019) find bisexuals (34.1%) are less likely vaccinate against influenza than heterosexuals (48.5%) and gays/lesbians (43.8%); and gays/lesbians report higher influenza vaccination than heterosexuals. Little work has jointly considered race/ethnicity and sexual orientation, although qualitative work done by Padilla and colleagues (2019) showed that among Hispanic members of the LBGTQIA, respondents reported skepticism about contracting the flu from the vaccine itself. Altogether, mixed evidence is found for racial/ethnic sexual minorities flu vaccination.

## Data and Methods

In this paper, I draw on multiple state-years of data from the Behavioral Risk Factor Surveillance System (BRFSS), a nationwide telephone-based survey, administered annually, that collects state-level health data from non-institutionalized individuals aged 18 and older in the United States (CDC, 2021d). The BRFSS survey collects information on health behaviors, healthcare access, and preventative healthcare practices and utilizes a disproportionate stratified sampling method to select households with telephones in each state (CDC 2000–2022). It includes a core module asked yearly and fielded to all respondents, as well as optional modules states can elect to include on their questionnaires. States may also write and include their own questions.

### Analytic Sample

To construct my analytic sample, I limited the full sample based on several criteria. First, since sexual orientation is included only as an optional module question, I limit the sample to 45 US states (see appendix Table 4 in Appendix 1) that asked about sexual orientation at least once on their questionnaire between 2011 and 2020 (starting *n* = 2,147,089). Following, I limited this sample to persons with valid information on racial/ethnic identity (*n* = 2,116,438). Racial/ethnic identity is constructed using the BRFSS computed race/ethnicity measure, which draws from two questions: “Are you Hispanic or Latino?” and “Which one or more of the following would you say is your race?” Those who respond “multi-racial” (*n* = 50,878) or “Other” (*n* = 13,046) were excluded due to the lack of specificity on their racial identity (remaining *n* = 2,052,514). Next, I limited the sample to respondents with valid information on sexual orientation (*n* = 2,038,142). This sample limitation draws from the question “Do you consider yourself to be:” “Straight,” “Lesbian or Gay,” “Bisexual,” “Other”; those who responded as “Other” (*n* = 15,861) were excluded from analysis due to lack of specificity on their sexual identity. Finally, I limited the sample to persons with valid information on influenza vaccination; this resulted in a final analytic sample of *n* = 1,986,432 adults living in 45 US states/territories (see appendix Table 4).

To limit bias and manage item non-response, multiple imputations using Stata 15 with chained equation were used for all control measures, listed below (Allison, 2001). Following von Hippel (2007), respondents with missing values on the dependent variable (influenza vaccination) were included in the imputation, but then dropped from the analyses.

### Measures

Influenza vaccination status is the dependent measure. It is included in the core module and asked annually each year: “During the past 12 months, have you had either a flu shot or a flu vaccine that was sprayed in your nose?”, where 1 = yes and 0 = no.

My independent variables of interest are race/ethnicity identity and sexual orientation. In all analyses that follow, I consider these measures jointly, and it includes the following 18 groups: white heterosexuals (*n* = 1,561,936), white gay/lesbians (*n* = 24,637), white bisexuals (*n* = 24,363), black heterosexuals (*n* = 125,224), black gay/lesbians (*n* = 1928), black bisexuals (*n* = 2,78), Asian heterosexuals (*n* = 51,825), Asian gay/lesbians (*n* = 747), Asian bisexuals (*n* = 951), Native Hawaiian/Pacific Islander (hereafter referred to as NH/PI) heterosexuals (*n* = 9672), NH/PI gay/lesbians (*n* = 280), NH/PI bisexuals (*n* = 248), AI/AN heterosexuals (*n* = 30,837), AI/AN gay/lesbians (n = 492), AI/AN bisexuals (*n* = 705), Hispanic heterosexuals (*n* = 143,989), Hispanic gay/lesbians (*n* = 2761), and Hispanic bisexuals (*n* = 3459).

Following Andersen’s access to healthcare model (Andersen, 1995; Phillips et al., 1998), control variables are clustered into predisposing, enabling, and need-based groups. *Predisposing measures* include sex (1 = female, 0 = male, hereafter referred to as 1 = women, 0 = men), age at interview (18–35; 36–50; 51–64; and 65 +), whether a child is present in the household (1 = yes, 0 = no), marital status (married = 1, unmarried couple = 2, formerly married = 3, and never married = 4), employment status (1 = yes, 0 = no), and education (high school or less = 1, high school = 2, some college = 3, and college or more = 4).

*Enabling variables* include total household income (less than 25,000 a year = 1, 25,000–49,999 = 2, 50,000–74,999 = 3, and 75,000 +  = 4), whether or not one has missed care due to cost last year (1 = yes, 0 = no), and has a personal doctor (1 = yes, 0 = no).

Lastly, *need-based characteristics* include having exercised in the past 30 days (1 = yes, 0 = no), body mass index is overweight or obese (1 = yes, 0 = no), currently smokes (1 = yes, 0 = no), and has a chronic illness including cancer, asthma, diabetes, stroke, coronary heart disease, or heart attack (1 = any of these conditions, 0 = none).

### Analytic Plan

I begin with a descriptive analysis of the sample stratified by racial/ethnic and sexual orientation group. Following, logistic regression models are used to examine how race/ethnicity and sexual orientation jointly shape the odds of flu vaccination. I begin with a pooled analysis that regresses flu vaccination on race/ethnicity, sexual orientation, and race/ethnicity*sexual orientation. Three models are presented (Model 1 adjusts for predisposing characteristics; Model 2 adds enabling characteristics; and Model 3 adds need-based characteristics). Next, I stratify the sample by race/ethnicity and regress flu vaccination on sexual orientation, following the same model-building sequence described above. Altogether, this analysis plan allows me to assess, from various angles, the interconnections between race/ethnicity, sexual orientation, and flu vaccination among US adults.

## Results

### Sample Characteristics

Predisposing factors include demographic and social characteristics representing the likelihood for persons to need care. Turning to Table 1, *predisposing* factors generally show mixed patterns. Across all racial/ethnic groups, a high proportion of bisexuals identify as women, and on average, heterosexuals are older than sexual minorities. Gays/lesbians within all racial/ethnic groups report the lowest frequencies of having a child present in the household within respective groups, whereas highest frequencies varied between heterosexuals (Asian, NH/PI, and Hispanic) and bisexuals (black and Hispanic) depending on race/ethnicity. In terms of education, the most advantaged group were Asians, with all Asians reporting about 40% or above for having at least a college degree. Among those with the least advantaged education status, AI/AN bisexuals and Hispanics have the poorest outcomes. Roughly one in four AI/AN bisexuals reported an education status of less than high school. Among Hispanics, 35.4% of heterosexuals have a less than high school educational status (highest relative to all other heterosexuals), followed by 24.1% of bisexuals (second highest relative to all other bisexuals), and 16.2% of gays/lesbians (highest relative to all other gays/lesbians). Heterosexuals typically had high rates of reporting they were married at the time of interview, within all racial/ethnic groups, and white heterosexuals indicated the highest frequency of being married at 58%, whereas AI/AN heterosexuals reported the highest rates of being formerly married at 27.6%. Among NH/PI adults, gays/lesbians had the highest rate of being employed within their racial ethnic group, whereas among white, black, Asian, and Hispanic adults, bisexuals had the highest rate of employment.

**Table 1:** Sample characteristics (weighted proportions) by racial/ethnic identity and sexual orientation

	White NH	Black NH	Asian NH	NH/PI	AI/AN	Hispanic
Total *N* = 1,986,432	*N* = 1,610,936	*N* = 129,530	*N* = 53,523	*N* = 10,200	*N* = 32,034	*N* = 150,209
Received annual Flu vaccine						
Heterosexual	43.0	34.0	43.9	33.7	37.2	31.6
Gay or Lesbian	45.9	36.5	39.0	32.1	31.1	33.7
Bisexual	35.4	30.2	46.5	29.1	32.7	28.7
Predisposing characteristics						
Women						
Heterosexual	51.7	54.4	50.5	49.5	49.7	50.0
Gay or Lesbian	39.9	46.2	36.9	49.2	44.1	35.5
Bisexual	67.5	70.9	62.0	57.7	65.8	65.6
Age 65 +						
Heterosexual	26.4	17.5	12.1	8.5	16.8	9.4
Gay or Lesbian	14.3	4.9	6.8	2.6	6.5	3.7
Bisexual	8.2	4.9	4.2	2.3	7.1	3.6
Child in household						
Heterosexual	31.0	39.0	40.9	50.6	39.7	55.6
Gay or Lesbian	15.1	26.8	16.9	39.9	25.1	29.6
Bisexual	34.1	44.4	39.0	42.9	42.5	48.2
Less than high school						
Heterosexual	7.8	14.9	4.5	11.6	19.0	35.4
Gay or Lesbian	5.4	11.4	2.9	12.7	15.1	16.2
Bisexual	10.3	12.3	4.9	10.6	25.2	24.1
High school						
Heterosexual	28.1	30.9	16.8	34.9	33.5	27.7
Gay or Lesbian	20.1	32.8	22.8	39.4	31.1	28.4
Bisexual	27.3	31.0	23.2	30.5	25.1	28.4
Some college	37.8	40.4	32.5	42.5	35.4	34.5
Heterosexual	33.4	34.4	25.4	31.7	33.0	24.6
Gay or Lesbian	33.0	35.2	28.6	29.7	39.1	35.1
Bisexual						
College and above						
Heterosexual	30.7	20.8	53.3	21.8	14.6	12.3
Gay or Lesbian	41.4	20.7	45.7	18.2	14.8	20.3
Bisexual	24.7	16.3		39.3	16.4	14.3
Married						
Heterosexual	58.0	33.9	56.1	48.3	41.2	46.0
Gay or Lesbian	26.3	10.6	17.2	20.1	18.1	17.6
Bisexual	27.5	12.6	32.8	24.3	24.8	18.6
Unmarried couple						
Heterosexual	3.4	3.3	1.9	4.3	4.9	9.5
Gay or Lesbian	16.6	7.8	7.5	6.8	11.4	13.2
Bisexual	11.8	5.7	6.0	7.2	10.3	10.9
Formerly married						
Heterosexual	21.2	25.6	9.6	15.7	27.6	16.7
Gay or Lesbian	10.9	11.1	12.3	10.9	15.5	8.3
Bisexual	15.7	13.9	4.9	11.6	19.9	12.8
Never married						
Heterosexual	17.3	37.3	32.4	31.7	26.4	27.8
Gay or Lesbian	46.2	70.1	63.0	62.1	55.0	60.9
Bisexual	44.9	67.7	56.4	56.9	45.0	57.7
Employed						
Heterosexual	67.2	67.4	84.9	82.7	65.2	82.1
Gay or Lesbian	74.5	78.4	86.6	94.3	71.2	85.6
Bisexual	82.1	78.9	91.0	87.2	47.0	86.4
Enabling Characteristics						
Income less than 25,000						
Heterosexual	20.1	39.9	17.8	31.8	43.8	45.1
Gay or Lesbian	22.3	44.4	24.1	33.8	48.8	40.1
Bisexual	33.7	49.6	33.6	48.9	54.3	49.4
Income 25,000–49,999						
Heterosexual	23.3	26.2	18.9	24.2	25.5	27.1
Gay or Lesbian	21.5	25.6	21.9	24.9	26.8	25.3
Bisexual	25.3	28.3	23.5	25.7	26.2	25.7
Income 50,000–74,999						
Heterosexual	17.1	13.0	15.0	13.9	12.1	10.9
Gay or Lesbian	15.3	12.4	15.2	13.8	10.6	14.4
Bisexual	14.0	9.0	10.8	10.1	8.6	9.6
Income 75,000 +						
Heterosexual	39.4	20.9	48.4	30.1	18.6	16.9
Gay or Lesbian	40.8	17.6	38.8	27.6	13.8	20.2
Bisexual	27.0	13.1	32.1	15.3	10.9	15.3
No HC due to cost						
Heterosexual	9.9	15.7	9.0	15.5	17.9	20.3
Gay or Lesbian	13.2	19.3	10.3	34.8	18.3	21.2
Bisexual	21.8	24.6	17.4	24.5	27.0	26.9
Has Insurance						
Heterosexual	92.5	86.5	91.9	83.7	87.4	70.9
Gay or Lesbian	90.9	81.0	88.5	77.1	89.0	76.7
Bisexual	87.9	82.1	88.0	73.6	86.1	75.2
Personal Doctor						
Heterosexual	84.0	79.3	79.1	73.9	73.5	63.1
Gay or Lesbian	82.9	74.2	69.1	71.0	67.2	67.4
Bisexual	72.9	70.5	73.3	59.8	71.2	62.2
Need-Based Characteristics						
Overweight or obese						
Heterosexual	64.9	73.9	42.3	69.4	71.5	71.4
Gay or Lesbian	62.2	64.5	39.0	66.2	63.6	61.3
Bisexual	60.3	69.2	33.9	58.6	67.8	64.9
Exercised in last 30 days						
Heterosexual	77.6	70.3	80.3	78.0	71.8	71.2
Gay or Lesbian	79.3	69.9	82.1	63.1	76.0	76.6
Bisexual	80.0	68.9	77.1	80.8	72.7	75.7
Currently smokes						
Heterosexual	16.3	18.0	7.8	18.9	28.3	12.1
Gay or Lesbian	22.3	28.7	17.0	34.3	31.0	20.6
Bisexual	26.1	25.3	8.1	35.7	39.8	20.7
Has a chronic illness						
Heterosexual	26.1	31.0	18.3	26.2	35.5	22.6
Gay or Lesbian	26.8	32.4	18.8	39.4	34.3	22.6
Bisexual	27.6	29.8	18.1	28.9	41.8	27.9

*NH/PI* Native Hawaiian/Pacific Islander, *AI/AN* American Indian Alaska Native AI/AN. *HC* Healthcare

Enabling characteristics (see Table 1) pinpoint factors allowing individuals to seek out healthcare, such as influenza vaccination. Black, NH/PI, AI/AN, and Hispanic adults report a modal income category of less than $25,000 annually, regardless of sexual orientation, suggesting great difficulty affording care. White and Asian bisexuals in their respective racial/ethnic groups report a modal income of less than $25,000 annually showing a bisexual disadvantage among these racial/ethnic groups. Black, NH/PI, AI/AN, and Hispanic adults regardless of sexual orientation, and white, Asian, and NH/PI bisexuals show constrained enabling profiles. White and Asian heterosexuals and gays/lesbians report advantaged statuses of earning $75,000 or more annually. For those who cannot afford healthcare due to cost, NH/PI gays/lesbians report the highest frequency, whereas Asian heterosexuals report the lowest. Hispanics generally report the lowest proportions of having health insurance (followed by NH/PI bisexuals), with heterosexuals having the lowest, whereas both white and Asian heterosexuals report the highest proportions of insurance. Hispanic bisexuals are also the least advantaged in having a personal doctor, followed by their heterosexual peers. White heterosexuals and gays/lesbians report the highest frequency of having a personal doctor.

Need-based characteristics position individuals for how likely they are to use healthcare services (i.e., influenza vaccination) based on factors positioning their health in riskier statuses. Those who reported the highest proportions of being overweight or obese were AI/AN heterosexuals, with Asian bisexuals reporting the lowest. NH/PI gays/lesbians report the lowest frequencies of exercising in the past 30 days, whereas their bisexual peers report the highest. Generally, select racial/ethnic minorities report higher proportions of being current smokers, such as black (gays/lesbians having the highest within racial/ethnic group), NH/PI (bisexuals having the highest within racial/ethnic group), and AI/AN (bisexuals having highest within racial/ethnic group). In general, white gays/lesbians and bisexuals report high proportions of being current smokers. Asians heterosexuals (7.8%) and bisexuals (8.1%) are the least apt to partake in this health behavior.

Turning to rates of chronic illness, at least 25% of black, NH/PI, and AI/AN adults, regardless of sexual orientation, report chronic illness, and rates increase depending on sexual orientation. White, AI/AN, and Hispanic bisexuals report the highest chronic health conditions within their racial/ethnic group (white: 27.6%, AI/AN: 41.8%% and Hispanic: 27.9%). Black, Asian, and NH/PI gays/lesbians (black: 32.4%, Asian 18.8% and NH/PI: 39.4%) report the highest proportions of chronic health conditions within their respective racial/ethnic groups. Generally, those showing profiles suggesting their profiles were most necessary of influenza vaccination were sexual minorities who were black, NH/PI, AI/AN, and Hispanic.

### Pooled Models

Table 2 shows odds ratios from logistic regression models predicting influenza vaccination among the pooled (full analytic) sample. After controlling for predisposing, enabling, and need-based characteristics (see Model 3), Asian (OR: 1.32***) and Hispanic (OR: 1.10***) heterosexuals report higher influenza vaccination relative to white heterosexuals, whereas black heterosexuals (OR: 0.80***) report lower vaccination. White gays/lesbians (OR: 1.39***) and white bisexuals (OR: 1.06*) report higher vaccination relative to their heterosexual peers. Select sexual racial/ethnic and sexual minorities vaccinate more than white heterosexuals, such black gays/lesbians (OR:1.29**), Asian bisexuals (OR: 1.90***), and Hispanic gays/lesbians (OR:1.37***) report higher influenza vaccination. Marginal evidence is present for suggests Asian gays/lesbians (OR: 1.42 +) for higher vaccination status relative to the reference group.

**Table 2:** Odds ratios from Logistic Regression Models Predicting Influenza Vaccination (Pooled Sample)

	Model 1	Model 2	Model 3
Race (Heterosexuals)			
White NH Ref	–	–	–
Black NH	0.81***	0.83***	0.80***
Asian NH	1.26***	1.30***	1.32***
Native Hawaiian/Pacific Islander	1.01	1.07	1.05
American Indian/ Alaska Native	0.97	1.02	1.01
Hispanic	0.97	1.15***	1.10***
Sexual Orientation (Whites)			
Heterosexual Ref	–	–	–
Gay/Lesbian	1.40***	1.38***	1.39***
Bisexual	1.03	1.07**	1.06*
Race × Sexual Orientation			
White × Heterosexual Reference	–	–	–
Black × Gay/ Lesbian	1.25*	1.30**	1.29**
Black × Bisexual	0.89	0.96	0.94
Asian × Gay/Lesbian	1.32	1.39 +	1.42 +
Asian × Bisexual	1.78***	1.90***	1.90***
Native Hawaiian/PI × Gay/Lesbian	1.12	1.21	1.20
Native Hawaiian/PI × Bisexual	0.98	1.16	1.16
American Indian/AN × Gay/Lesbian	0.93	0.97	0.96
American Indian/AN × Bisexual	0.95	1.01	1.00
Hispanic × Gay/Lesbian	1.25**	1.38***	1.37***
Hispanic × Bisexual	0.97	1.10	1.05

Model 1 adjusts for predisposing characteristics; Model 2 adds enabling characteristics to Model 1; and Model 3 adds need-based characteristics to Model 2

*p < 0.05, **p < 0.01, ***p < 0.001, + *p* < 0.10

**Fig. 1:**
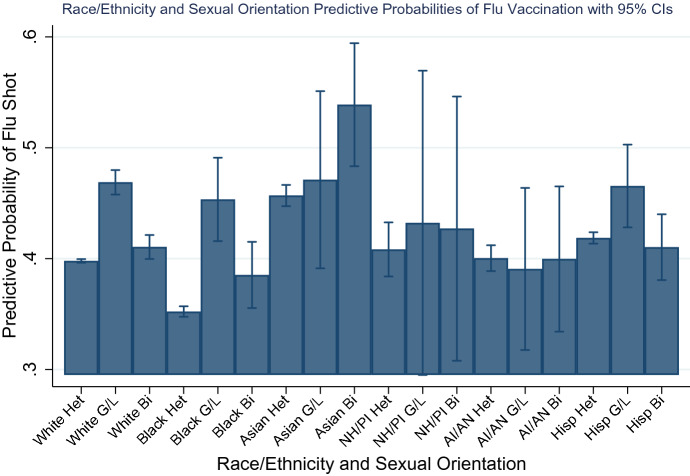
Predictive probabilities for influenza vaccination by race/ethnicity and sexual orientation

To facilitate a broader array of contrasts across racial/ethnic and sexual orientation groups, Fig. 1 graphs the interaction from Model 3 of Table 2 using predicted probabilities and shows some notable differences in the probability of flu vaccination across groups. The probability of vaccination hovers around 40% for heterosexuals within each racial/ethnic group, with whites being slightly below 40% and black heterosexuals experiencing the lowest rates around 35%. For black heterosexuals, this difference is significant relative to all other five racial/ethnic categories, and for whites, lower vaccination outcomes are significant compared to Asian and Hispanic heterosexuals. AI/AN heterosexuals are disadvantaged in vaccination status relative to their Asian and Hispanic peers, whereas Hispanics and NH/PI heterosexuals are disadvantaged only relative to their Asian peers and Asian heterosexuals having the most statistically significant advantageous status among all heterosexuals. For gays/lesbians, no clear pattern emerges in racial/ethnic differences as all confidence intervals overlap, with the probability of vaccination for white, black, Asian, NH/PI, and Hispanic gay/lesbian adults over 40% and AI/AN under 40%. Influenza vaccination patterns among bisexuals provides interesting insights on racial/ethnic differences on this health behavior. The probability of vaccination for black, AI/AN, and Hispanic bisexuals is generally under 40%. Among white, Asian, and NH/PI bisexuals, however, predicted probabilities of influenza vaccination are much higher, approaching 60%. For Asian bisexuals, this difference is significant when contrasted against all other groups—except for NH/PI bisexuals. Some notable differences across racial/ethnic sexual orientation groups are that white gays/lesbians are more apt to vaccinate relative to black, NH/PI, AI/AN, and Hispanic heterosexuals, and black and Hispanic bisexuals.

### Race/Ethnicity Stratified Models

Table 3 replicates the modeling sequence discussed above, but separately for each racial/ethnic group. Among whites, gay/lesbian adults (OR: 1.37,***) and bisexuals (1.07**) have higher odds of flu vaccination relative to heterosexuals. Black adults see a similar trend, with gays/lesbians (OR: 1.52***) reporting influenza vaccination at higher odds than heterosexuals, and bisexuals report a marginal difference (OR:1.13 +), after controlling for all characteristics. Among Asians, bisexuals (OR: 1.41***) report higher odds of influenza vaccination relative to heterosexuals. For NH/PI and AI/AN adults, results show no statistically significant relationship in their respective groups in any models. Hispanic gay/lesbians (OR: 1.37***) see a statistically significant relationship in all three models relative to their heterosexual peers.

**Table 3:** Odds ratios from Logistic Regression Models Predicting Influenza Vaccination, Stratified by Racial/Ethnic Identity

	Model 1	Model 2	Model 3
White NH adults (N = 1,610,936)			
Heterosexual ref	–	–	–
Gay/ Lesbian adults	1.38***	1.35***	1.37***
Bisexual adults	1.03	1.08***	1.07**
Black NH (N = 129,530)			
Heterosexual Ref	–	–	–
Gay/Lesbian	1.51***	1.51***	1.52***
Bisexual	1.10	1.13 +	1.13 +
Asian NH adults (N = 53,523)			
Heterosexual Ref	–	–	–
Gay/Lesbian	1.06	1.06	1.08
Bisexual	1.37*	1.41**	1.41**
Native Hawaiian/PI NH adults (N = 10,200)			
Heterosexual Ref	–	–	–
Gay/Lesbian	1.02	1.00	1.00
Bisexual	1.00	1.05	1.02
AI/AN NH adults (N = 32,034)			
Heterosexual Ref	–	–	–
Gay/Lesbian	0.90	0.88	0.87
Bisexual	1.01	1.01	0.98
Hispanic adults (N = 150,209)			
Heterosexual Ref	–	–	–
Gay/Lesbian	1.37***	1.33***	1.37***
Bisexual	1.00	0.99	0.99

Model 1 adjusts for predisposing characteristics; Model 2 adds enabling characteristics to Model 1; and Model 3 adds need-based characteristics to Model 2

*p < 0.05, **p < 0.01, ***p < 0.001, + *p* < 0.10

## Discussion

In this paper I apply an intersectional lens to ask whether and how flu vaccination varies across the intersections of racial/ethnic and sexual identity among a sample of US adults. Drawing on Andersen’s (1995) model of health services use, I also examine whether predisposing, enabling, and need-based factors contribute to patterns of intersectional immunity seen among BRFSS participants. In doing so, this paper contributes to existing scholarship in four ways. First, it corroborates previous studies of vaccination, finding lower flu vaccination among black adults relative to whites (Lu et al., 2014; Quinn et al., 2017); sexual minorities vaccinate at higher rates than heterosexuals (see Jones et al., 2016); and gays/lesbians vaccinate at higher rates than heterosexuals and bisexuals, with bisexuals reporting lower vaccination relative to gays/lesbians (see Srivastav et al., 2019). Second, it demonstrates how sexual orientation complicates established patterns between race/ethnicity and vaccination (e.g., influenza vaccination is racially stratified among heterosexuals, with patterns variable among gays/lesbians and bisexuals). Third, the findings pinpoint the intersectional identities most in need of influenza vaccination outreach efforts. Lastly, this paper analyzes groups that have often been left out of the literature due to small sample sizes, such as NH/PI and AI/AN heterosexuals and sexual minorities.

Beginning with patterns this study corroborates, I turn to black adults. Distinct differences between white and black adults for influenza vaccination status have been documented in various scholarship (Lu et al., 2014, Quinn et al., 2017; Quinn et al., 2018) with black adults having pronounced disadvantages in vaccination. My findings replicate this pattern—but only among heterosexuals. Among black heterosexuals, influenza vaccination odds remain low relative to both white heterosexuals, black gays/lesbians, and they report lower predictive probabilities of influenza vaccination relative to all heterosexuals. These patterns were present after adjustment for a variety of predisposing, enabling, and need-based characteristics, suggesting other factors unmeasured in this study could drive this finding. Within racial group differences among black adults are especially perplexing, as bisexuals typically report worse health outcomes relative to both heterosexuals and gays/lesbians (see Gorman et al., 2015; IOM, 2011; Schick & Dodge, 2012; Tuthill et al., 2020), but in the case of influenza vaccination, heterosexuals are the most disadvantaged. This emphasizes heterosexuality deserves more critical attention when studying sexuality. My study suggests there is something specific about heterosexuality in combination with black identity is driving lower vaccination. As previously discussed, black adults face many barriers to vaccinate against influenza, such as not being offered the vaccine (Travers et al., (2018) denying the vaccine due to mistrust and lower vaccine literacy (Chen et al., 2007; Quinn et al., 2016; Quinn et al., 2017; Quinn, 2018), and, in general, a lack of access to healthcare and quality healthcare due to racism (Phelan & Link, 2015). Research has also found higher proportions of racial consciousness led to lower vaccination rates among black adults (Quinn et al., 2017) and general worry about adverse effects of the vaccine (Freimuth et al., 2017). But most studies examine the lower rates of influenza among all black adults (e.g., Lindley, 2006; Lu et al., 2014) and do not simultaneously consider sexual orientation. Most studies examining black heterosexuals health examine HIV-related topics among black men (see Bowleg & Raj, 2012; Bowleg, Burkholder, Massie, Wahome, Teti, Malebranche, and Tschann, 2013) or are related to men’s sexual health, but little research examines black heterosexuality explicitly. Although research exploring black heterosexuals primarily focuses on men’s sexual well-being, scholars such as Lisa Bowleg and colleagues (2013) found having a one privileged identity aspect (i.e., heterosexuality) may not be as protective if their racial identity has been subjected to consistent racial oppression rooted in anti-black racism. Bowleg and colleagues pinpoint for black men, narratives of racism and poverty powerfully affect their health outcomes. Furthermore, black adults may underutilize healthcare if they perceive discrimination through their life course (Burgess et al., 2009). Among black heterosexuals, experiencing or anticipating discrimination at places administering healthcare, may be a driving force of their lack of influenza vaccination, as racial identity is their most salient identity.

Findings replicates prior work that gays/lesbians vaccinate more than heterosexuals and bisexuals (Srivastav, et al., 2019) but this was observed only among white gays/lesbians (relative to heterosexuals and bisexuals) Hispanic gays/lesbians (relative to their heterosexual peers) and black gays/lesbians (relative to heterosexual peers). Among white gays/lesbians, findings could be related advantaged SES, as most earn more than $75,000 annually and have a college degree or more (See Table 1). Additionally, roughly one-third of white gays/lesbians report having a chronic health condition, which may be a motivating factor to vaccinate against the bodily costs of influenza. This finding tracks to previous explanations of influenza vaccination among LGB adults and may represent why white gay/lesbian vaccination is higher, but this explanation does not entirely explain higher vaccination among black and Hispanic gays/lesbians. Black and Hispanic gays/lesbians report constraining need-based and enabling profiles (i.e., they both report a modal educational status of completing high school and report modal incomes of less than $25 k annually). However, their need-based characteristics may contribute to why black and Hispanic gays/lesbians are more apt to vaccinate relative to heterosexuals. Within racial group, they report relatively high frequencies of being current smokers (roughly 1 in 5 are current smokers among both groups) having higher rates of chronic illness, and report relatively high rates of being overweight or obese (64.5% of black gays/lesbians report and 61.3% of Hispanic gays/lesbians) and high rates of experiencing a chronic illness (see Table 1). These vulnerable health profiles suggest influenza vaccination may be critical to curb the deleterious effects of influenza illness among black and Hispanic gays/lesbians. However, this does not fully paint the picture of higher vaccination status among black and Hispanic gays/lesbians, as other groups report worse health profiles but do not vaccinate at higher rates. Indeed, their profiles suggest that they are at risk for experiencing the deleterious bodily and financial effects of an influenza illness, but it is important to also consider their historical relationship with healthcare access (Poteat, 2021). Vaccinating against influenza is an agentic option black and Hispanic gays/lesbians can partake in to mitigate risk of illness in an otherwise constraining structures of racism and homophobia that operate in tandem to allocate privilege and power to those who are not racial/ethnic and sexual minorities. In addition, it may be important to vaccinate the health of not only themselves, but their community and loved ones as well. Others have documented the importance of community, whether it be chosen or inherent (i.e., biological family), and its importance for both black and Hispanic gays/lesbians (Harris, Battle, & Pastrana, 2018). Work examining COVID-19 vaccinations found that among Latino sexual minority men, altruism worked as an important factor to increase COVID-19 vaccination (Weinstein et al., 2022). For black and Hispanic adults, their advantageous influenza vaccination status could reflect both a positive belief of taking care of oneself and community and the reality of taking extra steps to prevent illness in otherwise constraining social, political, and economic environments.

The last finding corroborating previous studies is bisexual disadvantage for influenza vaccination coverage. However, bisexual disadvantage within racial group was only found among whites and only relative to gay/lesbian peers (see Fig. 1). Low vaccination among white bisexuals tracks onto previous explanations of low vaccination among bisexuals, with their profiles being economically vulnerable (Srivastav et al., 2019). In this current study, white bisexuals within their racial group report poor enabling profiles relative to heterosexuals and gays/lesbians. Their modal income category is less than $25,000 annually (whereas heterosexuals and gays/lesbians report categories $75 k + annually), additionally they have the highest frequencies of not being able to afford healthcare, the lowest rates of both health insurance and having a personal doctor. Black and Hispanic bisexuals also saw a disadvantage, but this was present relative to gay/lesbian whites, meaning that they experienced disadvantage across race/ethnicity and sexual orientation axis, in which I detail more explanations for their experience in the third contribution of this study.

This study also shows how both sexual orientation and race/ethnicity interplay with one another to complicate previous patterns of influenza vaccination. On the one hand, sexual orientation complicates established patterns between race/ethnicity and vaccination (e.g., influenza vaccination is more racially stratified among heterosexuals, with patterns more variable among gays/lesbians). On the other hand, race/ethnicity complicates established patterns between sexual orientation and vaccination, with Asian bisexuals vaccinating more than heterosexual peers. Previous scholarship has found evidence for a bisexual health disadvantage relative to heterosexuals and gays/lesbians (Gorman et al., 2015; IOM, 2011; Schick & Dodge, 2012) as well as lower rates of influenza vaccination among bisexuals (Srivastav et al., 2019). Thus, we would expect bisexuals would have some unfavorable influenza vaccination outcomes. However, the current paper shows only a few select groups show disadvantage in bisexuals vaccination status in their racial/ethnic group– specifically among whites. Findings show among Asians, bisexuals are actually more likely to vaccinate against influenza relative to heterosexuals. Indeed, relative to white heterosexuals, Asian bisexuals report higher vaccination status (See Fig. 1). Looking at the descriptive profiles of Asian bisexuals (see Table 1), their socioeconomic and health profiles might motivate them to vaccinate against influenza.

Asian bisexuals might vaccinate against influenza to prevent against both financial and bodily costs of influenza. Their income status (modal income of less than $25 k annually) and health status (highest rates of being overweight or obese and chronic health conditions within racial/ethnic group) may be factors motivate in order to curb the negative impacts of influenza. Moreover, Asian bisexuals in this sample have generally decent health behaviors relative to other groups, with 6.8% report current smoking (the lowest in the entire sample) and 82.4% indicate exercising in the past 30 days (the second highest in the sample). These profiles underscore that this group partakes in relatively healthy behaviors despite having constraining enabling and need-based profiles. Additionally, previous studies examining Asian sexual and gender minorities (SGM) show they experience substantial barriers to healthcare, including stigma and minority status stress, implicit biases from healthcare providers, and clinic culture and policies that challenge healthcare access (Tan et al., 2016). Tan and colleagues also find Asian SGM adults fear discrimination within doctor’s offices and when receiving healthcare. While their study did not differentiate between Asian gays/lesbians and bisexuals, it does suggest Asian bisexuals may want to avoid interacting with medical settings, and thus utilize preventative measures in an attempt to lower their chances of both the financial, psychological (i.e., interacting with biased healthcare providers), and bodily costs of an influenza-related illness. In their intersectional location, Asian bisexuals see an advantage in influenza vaccination, but this should be interpreted with nuance. Their poor health and economic profiles suggest they *need* an influenza vaccination as they are intersectionality marginalized groups (Bowleg, 2021), and this could be due to experiences they jointly face as both bisexuals and racial/ethnic minorities.

This study’s third contribution pinpoints the location of intersecting marginalized identities most in need of influenza vaccination outreach efforts. As established, black heterosexuals experience poor vaccination status against influenza, and this study finds this is true relative to not only every other racial/ethnic heterosexual group, but also relative to their gay/lesbian peers. Indeed, heterosexuals in experienced lower vaccination relative to various gays/lesbians, as this was found among white and Hispanic adults as well. Again, this emphasizes heterosexuality should be given a more critical lens as to why an advantaged social and political status (i.e., heterosexuality) can lead to lower utilization of necessary preventative care, and especially for black adults this identity is not as protective for vaccinating against influenza.

Additionally, some evidence is present for NH/PI and AI/AN heterosexuals to vaccinate less across racial/ethnic and sexual orientation (relative to white gays/lesbians). For example, Fig. 1 points toward the complexity of intersectionality (Collins & Bilge, 2016; Misra et al., 2021) in which NH/PI and AI/AN heterosexuals report lower predicted probabilities of influenza vaccination relative to white gays/lesbians. For NH/PI and AI/AN heterosexuals, encompassing systems of settler colonization may be too powerful of a constraint to vaccinate against influenza relative to white gays/lesbians. Indeed, NH/PI and AI/AN share similar contemporary contexts and long-standing histories of settler colonization that have powerfully disrupted their lives and communities pre-colonization (Glenn, 2015). This, coupled with the case of heterosexual disadvantage in vaccination that has been found in this study, may lead to barriers to preventative healthcare (i.e., influenza vaccination).

Bisexuals were disadvantaged in influenza outcomes among their racial/ethnic group if they were white (relative to their gay/lesbian peers, see Fig. 1) or if they were black or Hispanic (relative to white gays/lesbians, see Fig. 1). Bisexuals typically face various barriers to accessing healthcare due to strained socioeconomic statuses, and white bisexuals descriptive statistics highlight several constraining circumstances in predisposing, enabling, and need-based characteristics. This finding suggests that factors such as homophobia among heterosexuals and biphobia among gays/lesbians shape poor vaccination outcomes among white bisexuals. For black and Hispanic bisexuals, this could largely be attributed to their joint identity status of being a sexual (importantly, bisexual) and racial/ethnic minority, as their predisposing, enabling, and need-based factors suggest they are more likely to have systemic constraints based off of their profiles, relative to white gays and/or lesbians. In addition, both bisexuals (IOM, 2011) and people of color have (Harris, Battle, and Pastrana, 2018) been excluded from certain aspects of power and privilege that have been afforded to white gays/lesbians. Although specific mechanisms of racial oppression may differ for black (i.e., anti-black racism) and Hispanic (e.g., anti-Hispanic/ Latino racism) bisexuals, shared histories of intersectional marginalization (Bowleg, 2021) may shape their ability to vaccinate against influenza. Ergo, for black and Hispanic bisexuals, they may face the same homophobia and biphobia as white bisexuals but have overlapping racial identities that has been subjected to multiple interlocking systems of oppression.

The last contribution of this study is its inclusion of analyzing groups that are often looked over in research studies, namely NH/PI and AI/AN heterosexuals and sexual minorities. A strength of the BRFSS’ robust data over the years allowed for a large sample of sexual minority NH/PI and AI/AN persons so analyses to be conducted. In other studies, these groups often have to be excluded due to their small sample sizes. For example, important work done by Agénor and colleagues (2019) examine HIV testing differences across race/ethnicity, gender, and sexual orientation, but authors had to exclude American Indians due to their small sample sizes. Although results yielded no significant findings within their repsective groups for NH/PI and AI/AN persons, BRFSS data allowed for further investigation pf joint relationships regarding racial/ethnic identity, sexual orientation, and influenza vaccination among mentioned overlooked groups.

### Limitations

The reader should keep in mind the following limitations when interpreting this study’s results. First, BRFSS does not ask questions surrounding nativity status, so results may not be capturing how immigration shapes racial disparities in influenza vaccination status. Additionally, information related to discrimination and bias in healthcare treatment is not available. These data also rely on a telephone-based sample, which includes only LGB adults who are comfortable to disclose their sexual orientation via surveys and are otherwise not out (IOM, 2011). Lastly, BRFSS data do not ask questions surrounding attitudes and beliefs toward vaccines, thus some of the findings cannot ascertain beliefs or attitudes about influenza vaccination.

## Conclusion

Overall, this study makes four contributions to existing literature using an intersectional perspective (Bowleg, 2012; Collins & Bilge, 2016; McCall, 2005) and drawing on Andersen’s (1995) model of healthcare utilization: (1) flu vaccination patterns are replicated among certain groups and corroborate previous influenza vaccination trends (e.g., racially stratified influenza vaccination status among heterosexuals), (2) certain groups challenge previous notions of influenza vaccination due to their intersectional location (e.g., Asian bisexuals), (3) identifying various intersectional groups in need of targeted influenza vaccination outreach efforts, and (4) studying groups that are often looked over in population health research (e.g., NH/PI and AI/AN sexual minorities). For example, healthcare providers and physicians should consider how medical mistrust among black heterosexuals may shape their lower odds of flu vaccination. Quinn (2018) emphasizes the large role public health agencies, healthcare providers, and the African American community play in vaccinating black adults. Evidence also shows physicians may not be providing strong enough recommendations for influenza vaccination, thus it is imperative healthcare providers and physicians give stronger recommendations for black adults regarding an annual influenza vaccination (Quinn, 2018). For sexual minorities, free influenza vaccination should be coupled with outreach and intervention by public health officials to administer information on the benefits of influenza in LGBT spaces and centers. This is critical as all racial/ethnic bisexuals report high frequencies of not affording healthcare costs. Importantly, physicians and healthcare administrators should be educated on LGBT health to create safe and inviting healthcare environments. In general, clinicians should familiarize themselves with how joint identity statuses shape vaccination among sexual minority persons of color and encourage continued high utilization of influenza vaccination among sexual minorities. Clinicians should encourage heterosexuals to partake in this preventative health behavior as it seems heterosexuals are less likely to vaccinate relative to their gay/lesbian peers. Lastly, policy makers and public health officials should advocate for free influenza vaccination clinics, and insurance companies should make flu vaccination cost free.

Influenza vaccination patterns may elucidate possible COVID-19 vaccination trends in future and how race/ethnicity and sexual orientation interplay to shape vaccination outcomes. Currently, COVID-19 vaccinations operate under different circumstances relative to influenza vaccination; importantly, COVID-19 vaccination is heavily politicized (Killgore et al., 2021), a situation influenza vaccination has not been subjected to. Thus, mechanisms for low influenza vaccination rates may better capture access to preventative healthcare, medical mistrust in communities of color from medical racism, and/or general apathy toward the influenza vaccine when compared to COVID-19 vaccination.

## Appendix 1

See Table 4.

**Table 4:** Interview States and years

Alaska	2011–2017, 2019–2020
Arizona	2011–2012, 2018, 2019
California	2011–2018. 2020
Colorado	2011–2013, 2015, 2017, 2019–2020
Connecticut	2015–2020
Delaware	2014–2019
Florida	2012, 2017–2019
Georgia	2015–2017, 2019–2020
Hawaii	2011–2020
Idaho	2011–2016, 2018–2020
Illinois	2013–2018, 2020
Indiana	2011, 2014–2017, 2020
Iowa	2012, 2014–2017, 2019–2020
Kansas	2014–2015, 2018–2020
Kentucky	2014, 2016
Louisiana	2014 2016–2020
Maine	2011–2015, 2017
Maryland	2014–2015, 2018–2019
Massachusetts	2011–2018, 2020
Michigan	2011–2014, 2020
Minnesota	2014–2020
Mississippi	2016–2020
Missouri	2015–2016, 2018
Montana	2011–2014, 2017–2020
Nevada	2014–2018
New Mexico	2011–2018, 2020
New York	2014–2020
North Carolina	2011–2020
North Dakota	2011–2012
Ohio	2011–2020
Oklahoma	2017–2020
Oregon	2011–2018
Pennsylvania	2014–2018
Rhode Island	2013–2020
Tennessee	2018–2019
Texas	2015–2020
Utah	2011–2020
Vermont	2014, 2016–2020
Virginia	2015–2020
Washington	2011–2020
West Virginia	2015, 2018–2020
Wisconsin	2011–2020
Wyoming	2014
Guam	2014, 2016–2020

## Appendix 2

See Table 5.

**Table 5:** Medians and ranges of response rates from BRFSS years 2011–2020

Interview year	Median response rate	Minimum, Maximum
2011	49.7	33.8, 64.1
2012	45.2	27.7, 60.4
2013	46.4	29, 60.3
2014	47.0	45.8, 60.1
2015	47.2	33.9, 61.1
2016	47.0	30.7, 65.0
2017	45.9	30.6, 64.1
2018	49.9	38.8, 67.2
2019	50.0	37.3, 73.1
2020	47.9	34.5, 67.2

Medians and ranges are from combined landline and cellphone interviews. Response rates were drawn from the following reports: 2011: (https://www.cdc.gov/brfss/pdf/2011_Summary_Data_Quality_Report.pdf), 2012: (https://www.cdc.gov/brfss/annual_data/annual_2012.html), 2013: (https://www.cdc.gov/brfss/annual_data/2013/pdf/2013_dqr.pdf), 2014: (https://www.cdc.gov/brfss/annual_data/2014/pdf/2014_dqr.pdf), 2015 (https://www.cdc.gov/brfss/annual_data/2015/pdf/2015-sdqr.pdf), 2016(https://www.cdc.gov/brfss/annual_data/2016/pdf/2016_ResponseRates_Table.pdf), 2017 (https://www.cdc.gov/brfss/annual_data/2017/pdf/2017-sdqr-508.pdf), 2018 (https://www.cdc.gov/brfss/annual_data/2018/pdf/2018-sdqr-508.pdf), 2019(https://www.cdc.gov/brfss/annual_data/2019/pdf/2019-sdqr-508.pdf), 2020 (https://www.cdc.gov/brfss/annual_data/2020/pdf/2020-sdqr-508.pdf).
